# Cervical intraepithelial neoplasia grade 3: development during pregnancy and postpartum

**DOI:** 10.1007/s00404-022-06815-7

**Published:** 2022-10-22

**Authors:** Frederik A. Stuebs, Franziska Mergel, Martin C. Koch, Anna K. Dietl, Carla E. Schulmeyer, Werner Adler, Carol Geppert, Arndt Hartman, Antje Knöll, Matthias W. Beckmann, Paul Gass, Grit Mehlhorn

**Affiliations:** 1 Department of Gynecology and Obstetrics, Erlangen University Hospital, Comprehensive Cancer Center Erlangen-European Metropolitan Area of Nuremberg (CCC ER-EMN), Universitätsstrasse 21–23, 91054 Erlangen, Germany; 2 Department of Gynecology and Obstetrics, Ulm University Hospital, Comprehensive Cancer Center Ulm (CCCU), Ulm, Germany; 3 Department of Medical Informatics, Biometry and Epidemiology, Friedrich Alexander University of Erlangen-Nuremberg, Erlangen, Germany; 4 Institute of Pathology, Comprehensive Cancer Center Erlangen-European Metropolitan Area of Nuremberg (CCC ER-EMN), Erlangen University Hospital, Erlangen, Germany; 5 Institute of Clinical and Molecular Virology, Erlangen, Germany

**Keywords:** High-grade squamous intraepithelial lesion (HSIL), Cervical dysplasia, Pregnancy, Treatment, Mode of delivery

## Abstract

**Purpose:**

The aims of the present study were to evaluate the development of untreated cervical intraepithelial neoplasia (CIN) 3 during pregnancy and to assess persistence, progression, and regression rates postpartum to identify factors associated with regression.

**Methods:**

In a tertiary gynecology and obstetrics department, a total of 154 pregnant women with CIN 3 were treated in the dysplasia unit. The follow-up findings were analyzed retrospectively on the basis of histological, cytological, and human papillomavirus (HPV) testing of 154 pregnant women confirmed as having CIN 3 in colposcopically guided biopsies.

**Results:**

The rates of persistence, regression, and progression of CIN 3 in these women were 76.1%, 20% and 3.2%, respectively. Data for the delivery mode was available for 126 women. The rate of regression was almost twice as high with vaginal delivery as with cesarean section, at 27.4 vs. 15.2%, whereas the rate of progression was lower with vaginal delivery, at 2.7 vs. 6.5%.

**Conclusion:**

The rate of persistence of CIN observed in this study is comparable to that reported in other studies. The study provides strong evidence for greater regression among women who have vaginal deliveries. Careful work-up is recommended postpartum for this group of women in order to rule out persistent CIN 3 or invasive disease.

## What does this study add to the clinical work

**Table:** 

A conservative treatment of CIN 3 during pregnancy is safe. Careful postpartum evaluation is necessary regardless of the route of delivery.	

## Introduction

Cervical cancer is the fourth most common cause of deaths related to gynecological cancer among women in Germany. Among pregnant women, it is the most common malignant disease [1]. The incidence of cervical cancer during pregnancy is between 0.02 and 0.9% [2]. Between 0.006 and 0.1 per 100.000 pregnant women are diagnosed with some degree of cervical intraepithelial neoplasia (CIN) during the course of pregnancy [3]. The highest incidence of high-grade intraepithelial lesions (HSILs; CIN 2/3) is found among women aged 25–35 and thus in the reproductive age group [4].

Colposcopy in pregnant women is more challenging than in nonpregnant women. Hormonal changes result in cervical hyperemia, hyperplasia of the endocervical glands, mucus overproduction, contact bleeding, and prolapsing vaginal walls [5, 6]. The combination of Pap testing and visual inspection with acetic acid, with a sensitivity of 85–93% for detecting cervical cancer, is mandatory in every examination of the cervix in a certified dysplasia unit [7]. The incidence of abnormal cervical cytology is comparable to that in nonpregnant women, although stromal decidualization leads to large nuclei that may be misinterpreted as dysplastic cells, particularly during the second half of pregnancy, leading to an overall higher rate of false-positive results [1, 5]. However, a colposcopy-directed biopsy has to be performed in order to establish a diagnosis of CIN 3 or cervical cancer [8].

There have been contradictory reports on the natural history of CIN 2/3 in pregnant women [1]. Origoni et al. reported the progression rate to invasive cervical cancer with 0.4% to be extremely rare [1]. By contrast, Coppolillo et al. observed a progression rate of 13.3%, with progression to microinvasive cancer occurring in four of 30 women [9]. Spontaneous regression postpartum is reported to occur in 16.7–69.3% of pregnant women with CIN 2/3 [5]. There is evidence that overexpression of sex hormones during pregnancy may facilitate cervical carcinogenesis by inducing squamous metaplasia in the transformation zone and by modifying the local immune system. The decrease in sex hormones after delivery may explain the increased regression [10]. The impact of the mode of delivery remains unclear [11].

The aim of the investigation was to further analyze the course of CIN 3 during and after pregnancy and evaluate whether the mode of delivery has any influence on the rate of regression.

## Methods

Between 1996 and 2020, 154 women with CIN 3 during pregnancy were seen at the certified Dysplasia Unit in Erlangen University Hospital, Germany. Abnormal cytology of the cervix was the most common reason for pregnant women being referred to the Dysplasia Unit.

Colposcopies in the department were performed in standardized conditions. The current Zeiss KSK 150 FC colposcope has been used since 2014. A conventional pap smear of the cervix, testing for hrHPV and application of 5% acetic acid to the cervix is the standard of care in the unit. In pregnant women, a cotton swab was used instead of an endocervical brush for the endocervical Pap smear and an HPV test instead of an endocervical brush. The classification of the Pap smear findings was carried out in accordance with the current Munich classification at its time.

Up to 2018, the hybrid capture test 2 (Digene^®^ HC2 HPV DNA Test, Qiagen) was used for detection of hrHPV (HPV-16, -18, -31, -33, -35, -39, -45, -51, -52, -56, -58, -59 and -68). HPV testing is currently carried out using the Abbott RealTi*m*e high-risk HPV assay on an Abbott *m*2000sp instrument. This assay separately detects HPV-16, HPV-18, and a pool of 12 additional hrHPV types (HPV-31, -33, -35, -39, -45, -51, -52, -56, -58, -59, -66 and -68).

When the examinations detect a major finding or a lesion suspicious for invasion according to the RIO 2011 classification [12], a colposcopy-directed biopsy was taken from the most suspicious part of the lesion using biopsy forceps. Expert pathologists did the cytology and pathology reviews. Decisions regarding further treatment were based on the cytology results, HPV testing, and the histological findings. Data for the women who were treated were collected retrospectively. The women were closely monitored during pregnancy with follow-up visits every eight weeks. Decision regarding mode of delivery was routinely made during the 34 weeks of pregnancy. The first follow-up was carried out eight weeks postpartum.

In cases in which delivery and surgery took place in Erlangen University Hospital, the women’s data were available in the electronic records. Women who gave birth or underwent surgery elsewhere were contacted retrospectively to supplement the data.

The aim of this retrospective analysis was to compare pregnant women who were diagnosed with CIN 3 of the cervix in relation to different variables (e.g., HPV high-risk positivity and mode of delivery). All women with abnormal Pap smear findings but without histologically confirmed CIN 3 were therefore excluded. Women for whom postpartum histological findings were not available were also excluded. Regression of lesions was defined as a lower-grade lesion being detected in the postpartum period (8 weeks after delivery) in comparison with the first examination. Persistent disease was defined as CIN of the same grade as at the initial diagnosis being found in the final operation. Disease progression was defined as histological evidence of a higher CIN grade or cancer at the postpartum surgery [e.g., Large Loop Excision of the Transformation Zone (LLETZ) or hysterectomy] in comparison with the initial consultation.

### Statistical analysis

The Wilcoxon signed rank test was used to compare histology during pregnancy with histology after pregnancy. The influence of the mode of delivery was examined in subgroup analyses. Mann–Whitney *U* tests were used to compare histology between the subgroups. The significance level was set to 0.05 and all statistical analyses were carried out using the R statistical package, version 4.0.3[13].

## Results

A total of 154 pregnant women for whom complete postpartum follow-up data were available were treated in the certified dysplasia unit at Erlangen University Hospital. Their median age at first visit was 31 years (range 17–43 years) and the average gestational week at first visit was 11.5 weeks (range 6–40 weeks). All patients had lesions that were classified as CIN 3 antepartum. The vast majority of biopsies were taken during the first visit of the women. No severe complications after biopsy were observed e.g., strong bleedings or abortion, preterm delivery. The week of pregnancy at birth was documented for 110 women. Seven cases of abortion and fourteen cases of preterm delivery were documented. None of these cases was associated to the examination during colposcopy. Twenty three women delivered past delivery date. The overall rate of persistence of the lesions was 76.1% (118/154). The rate of regression was 20% (31/154) and the rate of progression was 3.2% (5/154). The difference between the antepartum and postpartum histological findings was statistically significant (Table 1).

**Table 1:** Antepartum and postpartum histological findings (Wilcoxon signed rank test: *P* < 0.001)

Antepartum	Benign	CIN 1	CIN 2	CIN 3	Carcinoma
CIN 3 (*n* = 154)	11 (7.1%)	12 (7.7%)	8 (5.2%)	118 (76.1%)	5 (3.2%)

*CIN* cervical intraepithelial neoplasia

Data on the mode of delivery were available for 126 women (83.3%) (Table 2). For 28 women, the mode of delivery was not documented and they could not be contacted. Seventy-three women had vaginal deliveries and 46 had cesarean sections. The rates of regression were 27.4% (20/73) after vaginal delivery and 15.2% (7/46) after cesarean section. The rates of persistence were 69.9% (56/83) after vaginal delivery and 78.3% (41/55) after cesarean section; after vaginal delivery, the rate of progression was 2.7% (2/73) and after cesarean section it was 6.5% (3/466/55) (Table 2). For 42/46 (91.3%) women who underwent cesarean section the gestational age was documented. Nine women had preterm cesarean section. One woman had this because of colposcopic findings suspicious for cancer, which was confirmed postpartum. The other women had a preterm delivery due to obstetric reasons. For 84 women pre and postpartum human papilloma virus-status is documented. In 83 women, the high-risk human papillomavirus persisted postpartum and in one women clearance of hrHPV was documented. None of the women had treatment during pregnancy. Eighty-six women had a conization postpartum, 11 women had simple hysterectomy postpartum and three women with carcinoma were treated with a PIVER type hysterectomy whilst the other two women with cervical cancer had a conization.

**Table 2:** Mode of delivery relative to rates of regression, persistence, and progression in 126 women (Wilcoxon signed rank test: *P* < 0.001)

Mode of delivery	Regression	Persistence	Progression
Abortion (*n* = 7)	1 (14.3)	6 (85.3%)	0
Cesarean section (*n* = 46)	7 (15.2%)	36 (78.3%)	3 (6.5%)
Vaginal delivery (*n* = 73)	20 (27.4%)	51 (69.9%)	2 (2.7%)

## Discussion

### Principal findings

This retrospective study analyzed 154 women with histologically confirmed CIN 3 during pregnancy. The overall rate of persistence of the lesions was 76.1% (118/154), the overall rate of regression was 20% (31/174), and the rate of progression was 3.2% (5/154). The role of the mode of delivery was also investigated. For vaginal delivery the rate of persistence was 69.9% (51/73) and 78.3% (36/46) after cesarean section; after vaginal delivery, the rate of progression was 2.7% (2/73) and after cesarean section it was 6.5% (3/46).

### Results in the context of what is known

The course of high-grade squamous intraepithelial lesions during pregnancy is a matter of controversy in the literature. The reported rates of persistence, regression, and progression differ significantly in the various studies. The majority of the published studies are retrospective, and prospective trials are extremely rare (see Table 3). The severity of the precancerous lesions studied also varies. In a retrospective study by Palle et al. including 142 pregnant women with CIN 1–3, the persistence rate was 47%, and the rates of regression and progression were almost equal at 25 vs. 28%, respectively [14]. In another retrospective study with a comparable size, including women with CIN 2/3, the regression rate was much higher at 69.3%, with a persistence rate of 26.85% and a progression rate of 3.9% [15]. In a prospective trial including 71 pregnant women with CIN 3 and 59 women with CIN 2 the results of regression, persistence and progression were: 33.1%, 54.6% and 12.3%, respectively [16]. For further data on other studies see Table 3.

**Table 3:** Summary of other studies including women with CIN during pregnancy and postpartum

Study	Country	Study period	*N*	CIN grade	Study design	Regression (%)	Persistence (%)	Progression (%)	MoD
Ackermann et al. 2006 [22]	Germany	1996–2004	77	CIN 3	Prospective	34.2	63.1	2.7	–
Coppola et al. 1997 [23]	USA	1987–1992	26	CIN 1–3	Retrospective	12	80	8	–
Coppolillo et al. 2013 [9]	Argentina	1989–2008	30	CIN 2–3	Retrospective	16.7	70.0	13.3	+
Grimm et al. 2020[5]	Germany	2011–2017	64	CIN 2–3	Retrospective	38	60	1.6	–
Karrberg et al. 2013 [16]	Sweden	2001–2009	163	CIN 1–3	Prospective	33.1	54.6	12.3	+
Mailath-Pokorny et al. 2016 [19]	Austria	2005–2010	51	CIN 1–3	Retrospective	56.9	39.2	3.9	–
Schuster et al. 2018 [11]	Austria	2010–2015	40	CIN 1–3	Retrospective	50	40	10	–
Serati et al. 2008 [10]	Italy	2003–2007	36	CIN 2–3	Prospective	47.3	52.7	0	–
Yost et al. 1999 [15]	USA	1995–1996	153	CIN 2–3	Retrospective	69.3	26.8	3.9	–
Own study	Germany	1996–2020	174	CIN 2–3	Retrospective	20.7	70	6.3	+

*MoD* mode of delivery—mode of delivery had no significant influence on the rate of regression,  + mode of delivery had a significant influence on the rate of regression

There have been various attempts to explain the increase in regression after vaginal delivery that has been observed in some studies. The cervical trauma that occurs during delivery may lead to an inflammatory process in the epithelium of the cervix, which may promote repair mechanisms. Another theory favors the transient ischemic changes that occur in cervical tissue during ripening as being responsible for lesion regression [1]. Cervical ripening and the passage through the birth channel may lead to the loss of dysplastic cells and therefore can support regression of dysplasia [5, 17]. Also transient ischemic changes during vaginal delivery may be responsible for an increased rate of regression compared to women who had cesarean section [1]. None of these theories has yet been confirmed, and the data published in the literature are contradictory. Schuster et al. observed no difference in rates of regression in 63 women with abnormal cervical smears during pregnancy, among whom 35 were diagnosed with CIN 2/3. The rates of regression were similar in the two groups (vaginal delivery vs. cesarean section) [11]. This contrasts with a retrospective review by Chung et al., who reported regression rates of 92.9% in women who delivered vaginally and 63.2% in women with cesarean section; persistence of lesions was higher for cesarean section 15.8% than for vaginal delivery 2.4%. The progression was also higher for women who underwent cesarean section 21.1% compared to 4.8% for vaginal delivery [18]. By contrast, the rate of regression in the present study was almost twice as high for vaginal delivery as for cesarean section, at 27.4 vs. 15.2%, and a higher rate of progression was observed in women who underwent cesarean sections, at 6.5 vs. 2.7%. The increased rate of progression in women who underwent cesarean sections may be partly explained by the fact that four of the five women with invasive carcinoma had colposcopic findings with suspected invasion before delivery. This was the reason for carrying out cesarean sections, as vaginal delivery should be avoided in these cases in order to prevent postpartum bleeding.

The effects of pregnancy on the HPV infection is not clear. The increased immunological tolerance during pregnancy may support the infection or at least reduce the effectiveness of the immune system [1]. Another suggested explanation for the increased rate of regression is the clearance of hrHPV after vaginal birth. In a retrospective study including 64 women with CIN 2/3 no HPV clearance postpartum was recorded in women with persistent or partially regressive histology findings, but in women with complete response 36.4% women presented with HPV clearance [5]. These data are in contrast to a second retrospective study where 51 pregnant women with CIN I-III were matched to a control group of non-pregnant women and the hrHPV-status was compared. For pregnant and non-pregnant women HPV clearance rates (95% CI) were 26.7% (0.72–2.10) and 38.9% (0.36–1.52), respectively [19]. In our own study, the antepartum and postpartum hrHPV status was documented in 84 women. hrHPV clearance was documented in one of the women. In contrast to the published literature, we only see hrHPV clearance in a small number of cases. The number of patients in our study is not large enough for further interpretation of HPV diagnostics. The role of HPV clearance remains unclear and further research in studies with large numbers of patients including type-specific HPV data and virus load are necessary.

Smoking is known to be the most significant environmental risk factor for CIN 2/3 (OR 2.6; 95% CI 1.7–4.0), and the effect is dose-dependent (*P* = 0.002) [20]. In a Swedish population-based case–control study, smoking was found to be the only independent risk factor. In a study by Fader et al. smokers were not more likely to have high-grade disease or to undergo a cervical procedure postpartum than nonsmokers, but were less likely to experience spontaneous regression of cervical dysplasia than their nonsmoking counterparts [21].

### Strengths and limitations

This study has certain limitations. Firstly, its retrospective nature has limited the availability of some information. The data for the study were obtained from the certified Dysplasia Unit, to which women are sent for initial evaluation and further recommendations, but a significant number of women did not return to the Unit after delivery and were lost to follow-up. Only women for whom histology findings before and after delivery were available were included in this study. This limits the number of women included and may involve a bias. Women who had unremarkable colposcopy findings postpartum were not regarded as requiring surgery and did not undergo biopsies. This very likely reduced the rate of regression, but was done to rule out any interobserver variability. If microinvasion had been ruled out, a significant number of women were treated with ablative laser surgery without excision to minimize the damage done to the cervix and reduce the risk of preterm delivery. These women did not have another sample taken from the cervix and where excluded from this study, and this further reduces the number of women included.

This is a comparably large study on CIN 3 during pregnancy. It provides important evidence on the characteristics of CIN 3 during pregnancy. A conservative treatment during pregnancy is save and vaginal delivery can be recommended.

### Implications for practice and future research

Although the rate of regression may be higher in pregnant women in comparison with nonpregnant women, careful follow-up is recommended postpartum vaginal delivery is safe in women who are diagnosed with cervical intraepithelial neoplasia 3 during pregnancy, and it may increase the rate of regression postpartum. Nevertheless, pregnant women should be seen in a specialized dysplasia unit after delivery to assess the rate of change and plan further treatment. The role of different types of the hrHPV in pregnant women is unclear and might play a role in the course of CIN during pregnancy. Also the week of delivery and the weight of the newborn might be of interest and should be investigated in further research.

## Conclusions

Conservative management for women with CIN 3 during pregnancy is safe, but careful postpartum evaluation is necessary regardless of the route of delivery. However, there is some evidence that vaginal delivery may increase the rate of regression. Antepartum evaluation should rule out invasive cervical cancer in order to avoid the risk of a poorer prognosis with vaginal delivery. If (micro-) invasion cannot be ruled out before delivery, a cesarean section should be performed.

## Abbreviations

ASC: Atypical squamous cell(s)
CIN: Cervical intraepithelial neoplasia
HC2: Hybrid capture test 2
HE: Hematoxylin–eosin
HSIL: High-grade intraepithelial lesion
HPV: Human papillomavirus
hrHPV: High-risk human papillomavirus
LSIL: Low-grade intraepithelial lesion
